# Quantification of Histotripsy Dosage Using Machine Learning-Enhanced Ultrasound Imaging Analysis: Correlation with Histological Outcomes

**DOI:** 10.1016/j.acra.2026.05.010

**Published:** 2026-06-10

**Authors:** Haowei Tai, Tejaswi Worlikar, Zhen Xu, J. Brian Fowlkes, Jiaqi Shi, Man Zhang

**Affiliations:** Department of Radiology, University of Michigan, Ann Arbor, Michigan (H.T., T.W., J.B.F., M.Z.); Department of Biomedical Engineering, University of Michigan, Ann Arbor, Michigan (Z.X., J.B.F.); Department of Pathology, University of Michigan, Ann Arbor, Michigan (J.S.)

**Keywords:** Histotripsy, Hepatocellular carcinoma, Tumor ablation, Ultrasound, Texture analysis

## Abstract

**Rationale and Objectives:**

Histotripsy is a noninvasive ultrasound therapy that mechanically disrupts target tissue through controlled acoustic cavitation. Clinically, a fixed histotripsy dose (number of pulses) is used for treating liver tumors, which does not account for tumor heterogeneity. Since histotripsy-induced damage can vary based on the tumor’s mechanical properties, there is a clinical need for a reliable and noninvasive method to measure the extent of cellular damage. Here, we present a quantitative, noninvasive, and image-based approach to evaluate histotripsy-induced tumor cellular damage by combining ultrasound texture analysis with machine learning and correlating these results with histology.

**Materials and Methods:**

Immunocompetent rats (n = 20) bearing orthotopic liver tumors were treated with varying histotripsy doses (20, 50, 100, and 200 pulses per location), covering the spectrum from sparse treatment to overtreatment. Pre- and post-histotripsy B-mode ultrasound images were obtained, and texture analysis was performed followed by feature reduction. Tumors were harvested post-treatment to quantify cellular damage (area covered by nuclear debris, intact nuclei, and necrosis scoring) via histology.

**Results:**

Strong correlation was observed between area covered by nuclear debris and first-order kurtosis (R^2^ = 0.9) and autocorrelation (R^2^ = 0.8). A random forest classifier trained on these features produced dose predictions that closely matched the administered values (R^2^ = 0.9, p < 0.05).

**Conclusion:**

Our results show that subtle texture changes in post-treatment vs. pre-treatment ultrasound images can serve as reliable indicators of dose-dependent tumor cellular disruption generated by histotripsy, as evidenced by their strong correlations with histological analysis. Moving forward, integrating real-time quantitative imaging feedback into clinical practice could help clinicians tailor histotripsy dosing more precisely for each patient.

## INTRODUCTION

Liver cancer poses a major clinical challenge in oncology globally (1). Current locoregional therapies (2–7) are often restricted by anatomical location, underlying comorbidities, and additional risk factors. Histotripsy is a promising noninvasive, non-ionizing, and non-thermal locoregional ultrasound ablation technique. It uses high-amplitude, microsecond-length ultrasound pulses to generate controlled acoustic cavitation microbubbles from endogenous nanometer-scale gas pockets in the target tissue, consequently disrupting its cellular structure (8,9). Preclinical studies and clinical trials have established the safety and efficacy of histotripsy for treatment of liver cancers (10–14), which led to U.S. Food and Drug Administration (FDA) clearance of histotripsy for the noninvasive treatment of primary and metastatic liver tumors (17,18). Currently, clinical histotripsy procedures use a single predefined dose (defined as the number of ultrasound pulses) for all liver tumor types, aiming to maximize tumor destruction. Recent *ex vivo* evidence suggests that histotripsy dose necessary for complete ablation of different human liver tumors likely depends on mechanical properties such as tissue stiffness, with higher pulses required for cholangiocarcinoma tumors compared to colorectal metastasis or hepatocellular carcinoma (15,16). This in turn affects the release of neoantigens and cascade of the immune response (17,18). These observations highlight the importance of personalizing histotripsy dose based on the mechanical and biological properties of each tumor. A primary barrier is the lack of a direct, noninvasive approach to measure the extent of tumor fragmentation. Clinically, B-mode ultrasound is used to guide histotripsy. However, visually examining B-mode ultrasound images alone is not enough to reliably distinguish between incomplete, complete, and excessive ablation, as subtle differences can be easily missed. While histological evaluation of excised tissues is the current gold standard in preclinical research, it is not practical to perform this kind of analysis on ablated tumors in human patients.

To address this gap, we have developed an advanced texture-analysis method to identify static imaging biomarkers from ultrasound that correlate with histologic tumor damage, which serves as a clear indicator of the level of histotripsy-induced cellular disruption. In the future, this approach may support personalized histotripsy protocols tailored to individual tumor characteristics, thereby improving therapeutic outcomes.

## METHODS

### Animal Model and Ethics

All procedures were approved by the Institutional Animal Care and Use Committee (IACUC) at the University of Michigan and performed in compliance with relevant guidelines and regulations. Male Sprague Dawley rats (n = 20) were injected with 10^6^ McA-RH7777 cells (ATCC^®^ CRL-1601^™^) suspended in 100 μL of serum-free media and Matrigel (1:1) into the liver lobes following a laparotomy procedure, as described previously (19). Liver tumors formed within 2 weeks and reached approximately 6 to 8 mm in diameter based on ultrasound measurements. Rats were then randomized into treatment groups (n = 5 per group).

### Histotripsy System and Treatment Protocol

**Figure 1.**
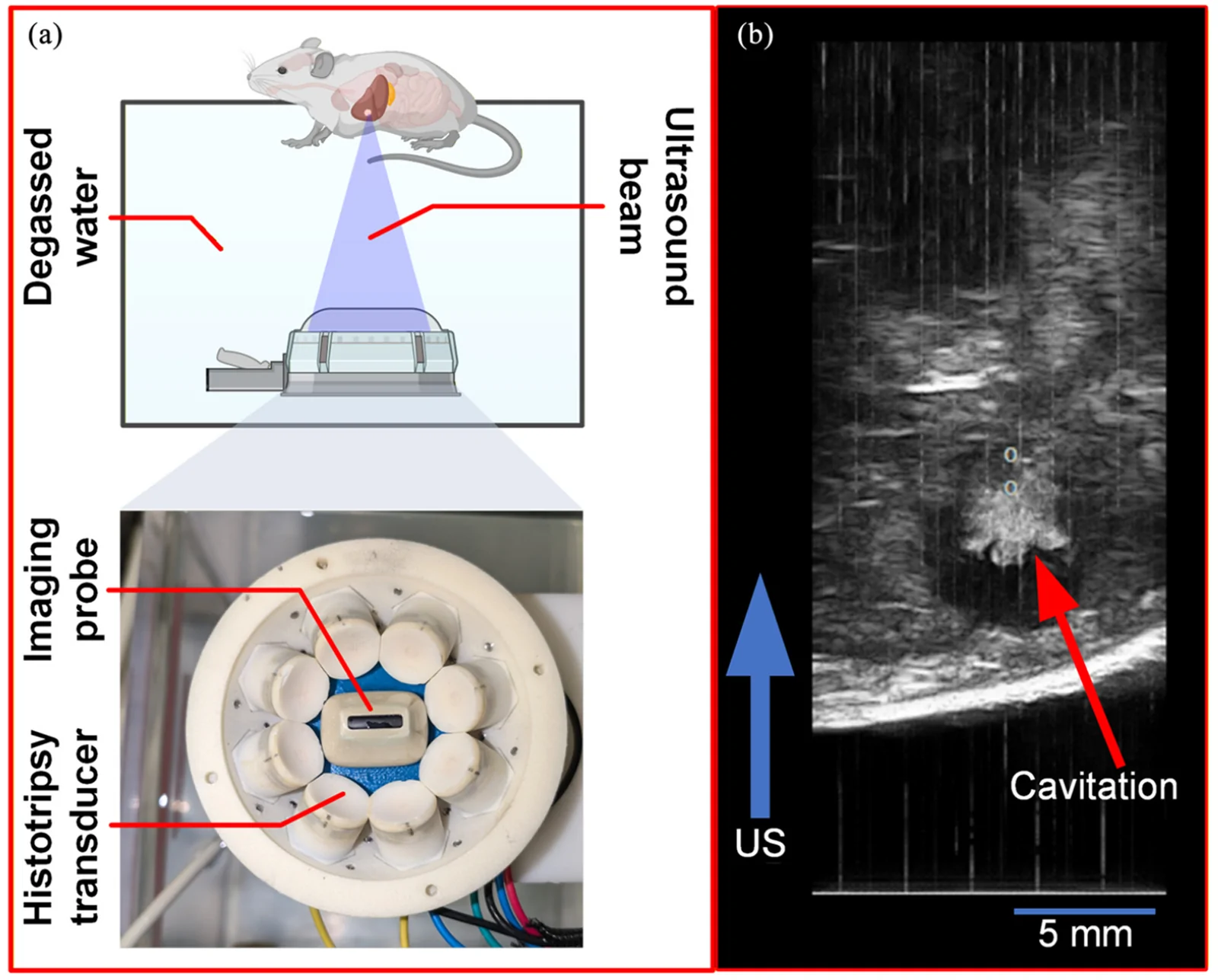
**(a)** The rodent histotripsy treatment system utilized an eight-element, 1 MHz therapy transducer delivering 1 cycle pulse at approximately 30 MPa negative pressure. A coaxially aligned 20 MHz imaging probe provided real-time ultrasound guidance and captured data for texture analysis to quantify changes in tissue microstructure. Both transducers were secured to a motorized positioning system and immersed in a tank of degassed water for acoustic coupling. The animal was positioned prone on a treatment bed, ensuring the targeted region remained submerged. **(b)** During histotripsy, a hyperechoic cavitation cloud forms (red arrow) and mechanically destroys tissue at the focal region. The blue arrow indicates the direction of propagation for the histotripsy field. Scale bar = 5 mm.

A custom-built, 8-element, 1 MHz histotripsy transducer with a focal distance of 32 mm was used (19). The histotripsy therapy transducer was driven by a 1-cycle pulse, generating an acoustic waveform with a single dominant negative pressure phase. The value of peak negative pressure in the tissue is estimated based on pressure measurements from a fiber optic hydrophone in the free-field as reported previously (19). Histotripsy doses were defined by the number of ultrasound pulses per location (ppl). Four different histotripsy doses were investigated in this study: 20 ppl, 50 ppl, 100 ppl, and 200 ppl delivered at pulse repetition frequencies of 20, 50, 100, and 200 Hz, respectively, with 0.7-mm spacing between grid locations and an estimated peak negative pressure > 30 MPa. Additionally, the setup included a three-axis motorized positioning system (for positioning the therapy and imaging transducers) and a water bath (with filtration, heating, and degassing) for acoustic coupling as described previously (19). Animals were anesthetized using inhaled isoflurane, and hair was shaved to ensure good ultrasound coupling. During histotripsy, animals were placed prone on the treatment bed such that the chest and abdomen were submerged in degassed water through an aperture in the bed. The histotripsy transducer focus was centered on the tumor region in the rat liver using real-time ultrasound guidance, Figure 1. Ultrasound imaging was performed to determine the extent of the tumor and the volume to be targeted was selected by setting the target dimensions. Next, a volume treatment grid consisting of target locations was generated, the motorized positioner moved the therapy transducer and the co-aligned 20 MHz imaging probe (UI-trasonix, Richmond, British Columbia, Canada) throughout the target volume to achieve volumetric ablation. Because safe treatment of the entire tumor volume was limited by tumor size, proximity to adjacent organs, and motion associated with free breathing, adequate treatment margins could not be maintained. Consequently, approximately 50–75% of the tumor volume was targeted using four different histotripsy doses. Tumors ranged from 7–10 mm in the largest dimension and the treatment times ranged from 5–15 min depending on the tumor volume treated.

### Pre- and Post-histotripsy Ultrasound Image Acquisition for Image Analysis

A GE LOGIQ^®^ E10 (GE Healthcare, Wauwatosa, WI) ultrasound system equipped with a linear L6–24 transducer was used to acquire tumor images both before treatment and immediately after histotripsy. B-mode image data were exported in DICOM format with corresponding scanner acquisition settings for offline processing and texture analysis. Then, a well-trained sonographer selected two to three post-treatment images per animal. In addition, one pre-treatment frame showing the largest tumor area was selected for each animal. The tumor and ablated regions were subsequently annotated on these images, and the resulting ultrasound imaging dataset was used for feature extraction and further analysis.

### Histology Slide Preparation and Analysis

Animals were euthanized and tumors were excised immediately post-treatment for histological analysis (n = 8; 2 from each group). Samples were fixed in 10% neutral-buffered formalin and submitted to the Tissue for Molecular Pathology Core Facility (Ann Arbor, MI, USA) for paraffin embedding and processing. Four-micrometer sections were sliced and stained with hematoxylin and eosin (H&E). Representative H&E slides (one central slice from each sample) were optically scanned and imaged at 20x magnification (Nikon Eclipse 50i). Five fields of view of the ablation zone were acquired from each sample. First, the percentage of area covered by nuclear debris (defined as intact nuclei and nuclear fragments) was quantified as a fraction of the total area in the field of view using color thresholding in ImageJ software (20). Next, intact tumor nuclei counts and the percentage of area covered by intact nuclei in the ablation zone were calculated using the open-source plugin StarDist (21) in ImageJ. Additionally, a clinical pathologist reviewed the histology slides (one central slice from each sample), and scored the level of necrosis on a scale from 0 to 5 (0 = 0%, 1 = 1–25%, 2 = 26–50%, 3 = 51–75%, 4 = 76–95%, 5 = > 95%) to quantify the area of tumor cell death. This score, which is often reported as a percentage of the total tumor volume, is key for evaluating post-treatment effects and prognosis.

### Texture Analysis and Machine Learning for Static Imaging Biomarkers

**Figure 2.**
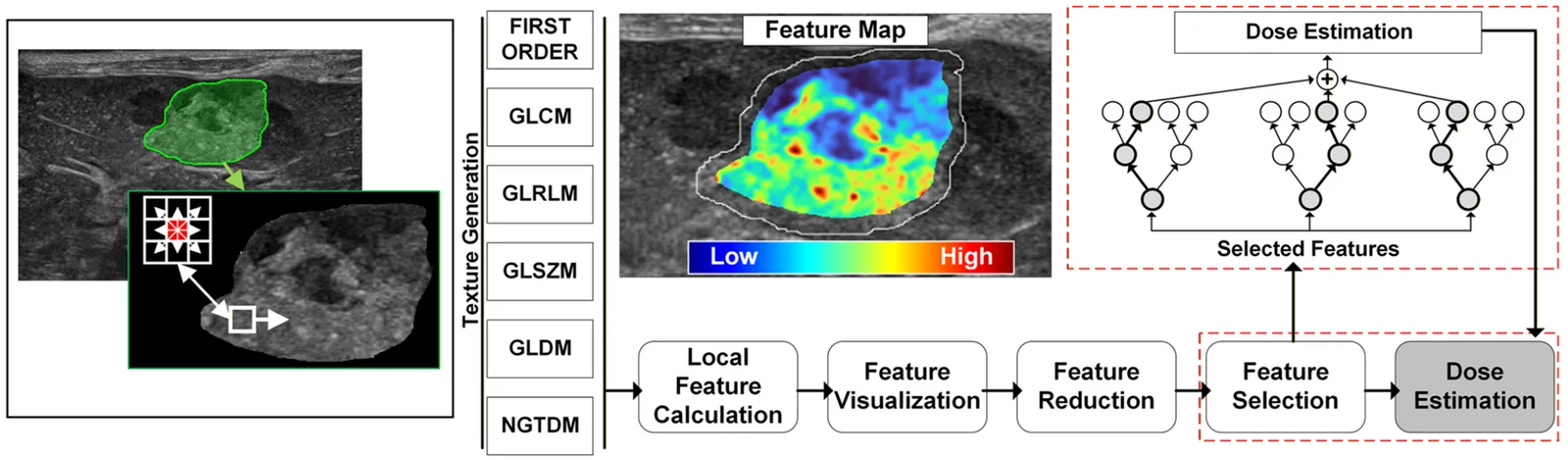
Schematic illustration of the texture analysis workflow. A 3×3 pixel sliding window was applied to capture local pixel interrelationships that might indicate microstructural tissue variations. First-order and higher-order textural measures were computed to provide a comprehensive view of pixel intensity differences, yielding 93 candidate features organized into localized feature maps. To reduce dimensionality, the PCC was used to remove redundant features. A subsequent selection step identified the top two features most strongly correlated with histological findings. Finally, the resulting feature subset was used to train a random forest classifier for histotripsy dose estimation (highlighted by the red dotted box). PCC, Pearson correlation coefficient. (Color version of figure is available online.)

Texture analysis was performed on the region of interest (ROI) identified by a single experienced observer (MZ) in each pre- and post-histotripsy ultrasound image to extract textural features indicative of microscale tissue changes. Based on our previous study (22,23), the open-source radiomics package (24) was used with a 3 × 3 sliding kernel to capture local intensity relationships among neighboring pixels (Fig 2). This approach produced feature maps that reflected spatial patterns related to underlying textural changes. A total of 93 features were extracted by combining first-order intensity statistics, Gray Level Co-occurrence Matrix (GLCM), Gray Level Run Length Matrix (GLRLM), Gray Level Size Zone Matrix (GLSZM), Gray Level Dependence Matrix (GLDM), and Neighboring Gray Tone Difference Matrix (NGTDM). For each animal, pre- and post-treatment ultrasound images were selected, and the tumor and ablated regions were annotated. Feature maps were generated, and feature values were calculated as the average across maps. Note that when multiple frames were selected for a single animal, the corresponding feature values were averaged across frames.

Feature reduction and selection followed a two-step strategy. First, variables were flagged as redundant if the absolute Pearson correlation coefficient (PCC) exceeded 0.8. One feature from each correlated pair was then removed, and the remaining features were normalized by expressing them as a percentage of their maximum values. Second, the reduced feature set was evaluated for nonlinear correlations with the percentage of nuclear debris measured by histology, and features exhibiting the strongest relationships (R^2^ > 0.8) were retained for further analysis.

A random forest classification model (25) was used with the extracted features and treatment dose to validate the feasibility of the feature extraction in capturing microstructural changes. The dataset was split into a training set (70%) and a testing set (30%). This ensemble-based approach aggregated outputs from multiple decision trees trained on bootstrap samples of the data.

### Statistical Analysis

Data were summarized as mean ± standard deviation (SD). Pearson correlation analysis was used to assess the relationship between static imaging biomarkers and histology. The relationships between histology-derived cellular damage and applied therapy dosage, as well as between static imaging biomarkers and histological measurements, were evaluated using nonlinear power models (26,27). Due to the limited sample size, formal hypothesis testing to confirm the power-model functional form or compare alternative model forms was not performed, and these analyses were used to describe potential associations rather than confirm a specific functional relationship. A Brown–Forsythe test was conducted to assess sample variance, followed by Analysis of Variance (ANOVA) and post hoc multiple comparisons using dose levels as factors to detect significant differences in the histological or imaging data. In addition, a paired t-test was performed to assess changes in the features from pre- to post-histotripsy within each dosage group. The correlation coefficient (R^2^) and p-value were computed to assess model reliability. A p-value of less than 0.05 was considered significant. Curve fitting analyses were performed using Prism 10 (GraphPad Software, San Diego, CA).

## RESULTS

### Texture Analysis

**Figure 3.**
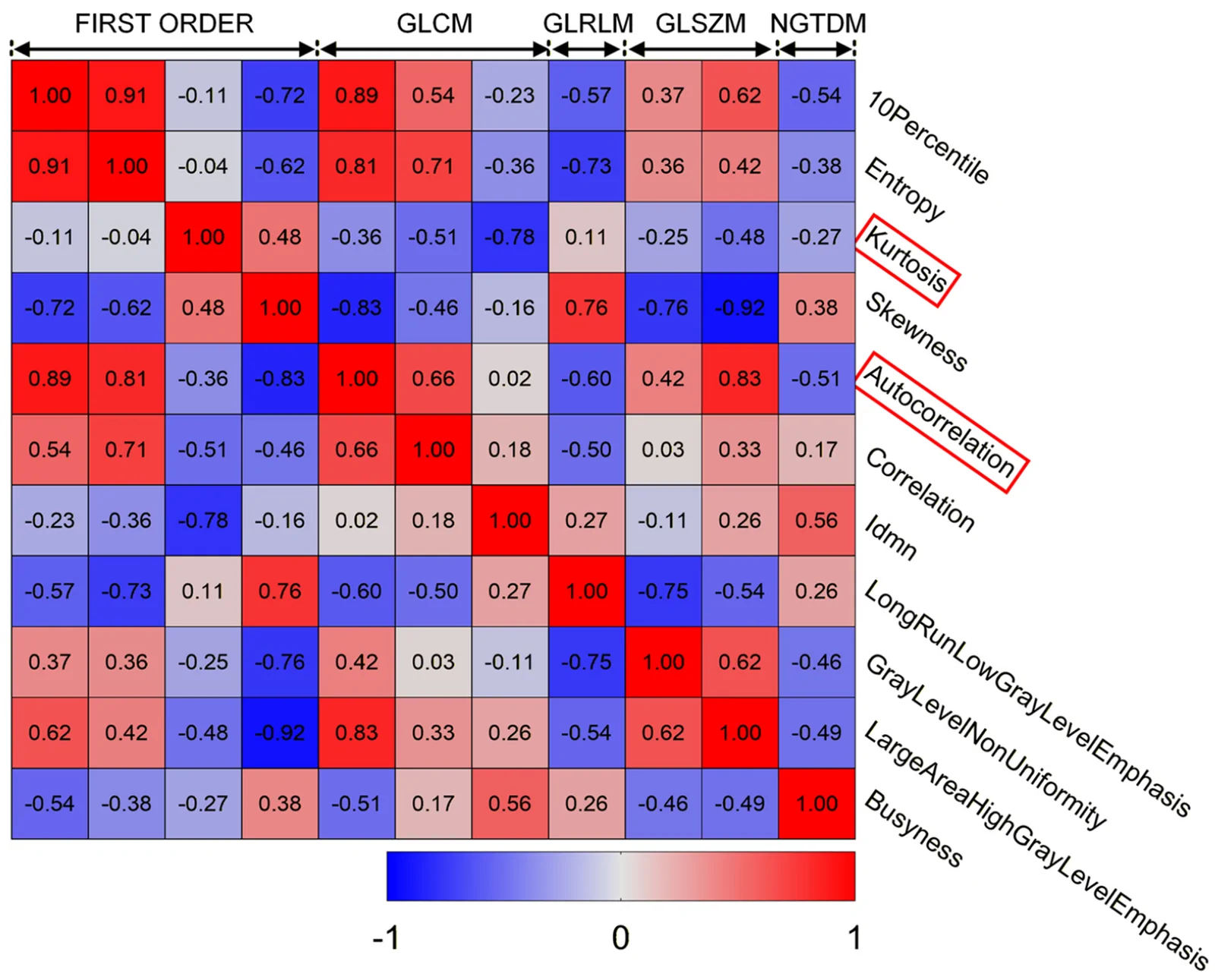
An absolute PCC threshold of 0.8 was used to remove one feature from each highly correlated pair, resulting in a reduced set of 11 features spanning first-order, GLCM, GLRLM, GLSZM, and NGTDM. Each cell of the correlation matrix is color-coded and annotated with its corresponding correlation coefficient. Subsequent feature selection further narrowed the set to two features based on their correlation with histological results (highlighted by red boxes and shown in red text in the legend) for tissue characterization: Kurtosis (from the first-order group) and autocorrelation (from GLCM). GLCM, Gray Level Co-occurrence Matrix; GLRLM, Gray Level Run Length Matrix; GLSZM, Gray Level Size Zone Matrix; GLDM, Gray Level Dependence Matrix; and MGTDM, Neighboring Gray Tone Difference Matrix; PCC, Pearson correlation coefficient.

The process of PCC comparisons eliminated redundant features to reduce the feature set. An absolute PCC threshold of 0.8 was used to remove one feature from each highly correlated pair, resulting in a reduced set of 11 features spanning first-order, GLCM, GLRLM, GLSZM, and NGTDM. The PCC values for these 11 features are shown in Figure 3. Subsequently, this reduced feature set underwent further selection based on correlations with nuclear debris quantification from H&E histology (described in Histological Analysis below). The goal was to select features strongly associated with histological findings, defined by a R^2^ greater than 0.8 based on nonlinear power regression. Ultimately, two features emerged as the most significant: Kurtosis from first-order analysis (R^2^ = 0.9), and Autocorrelation from the GLCM group (R^2^ = 0.8). These final two selected features are highlighted within red dot boxes along the diagonal in the correlation matrix shown in Figure 3.

**Figure 4.**
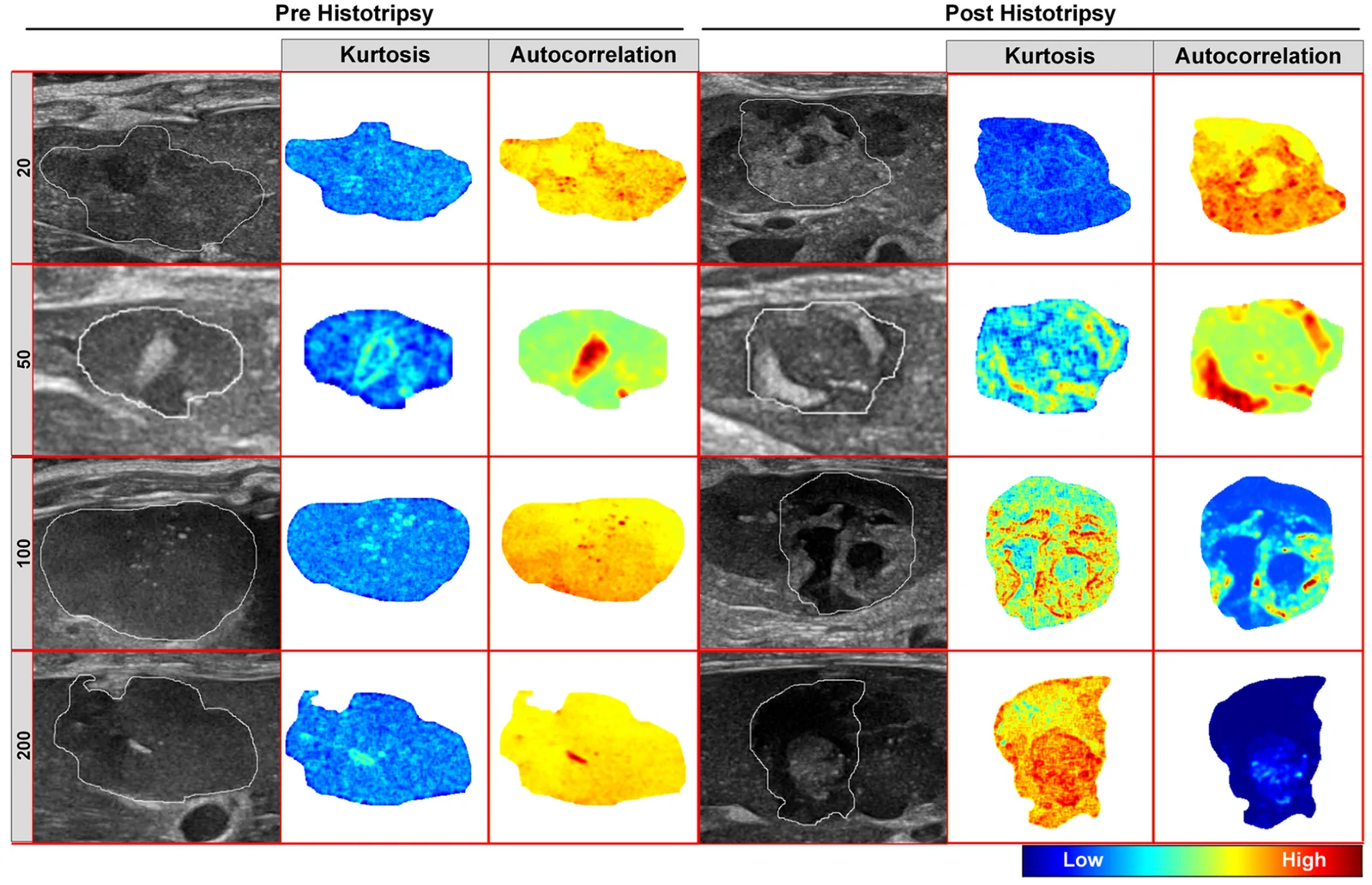
Examples of ultrasound texture-feature maps generated from a single region of interest (ROI) across histotripsy doses of 20, 50, 100, and 200 ppl, shown pre- and post-histotripsy. From left to right, the two displayed metrics are Kurtosis and Autocorrelation, arranged from top (lowest dose) to bottom (highest dose). Note that the same animal was used for the before-and-after comparison at each dose.

Figure 4 shows representative ultrasound images from different rats treated with increasing histotripsy doses (20, 50, 100, and 200 ppl, respectively), alongside corresponding texture maps of the two selected features. These maps reveal varied texture patterns across regions within the region of interest (ROI). Prior to histotripsy, the features show a similar appearance. Post-histotripsy, there is a progressive shift in color within these texture maps corresponding to the increase in treatment dose. Note that each rat served as its own control for the before-and-after comparison.

**Figure 5.**
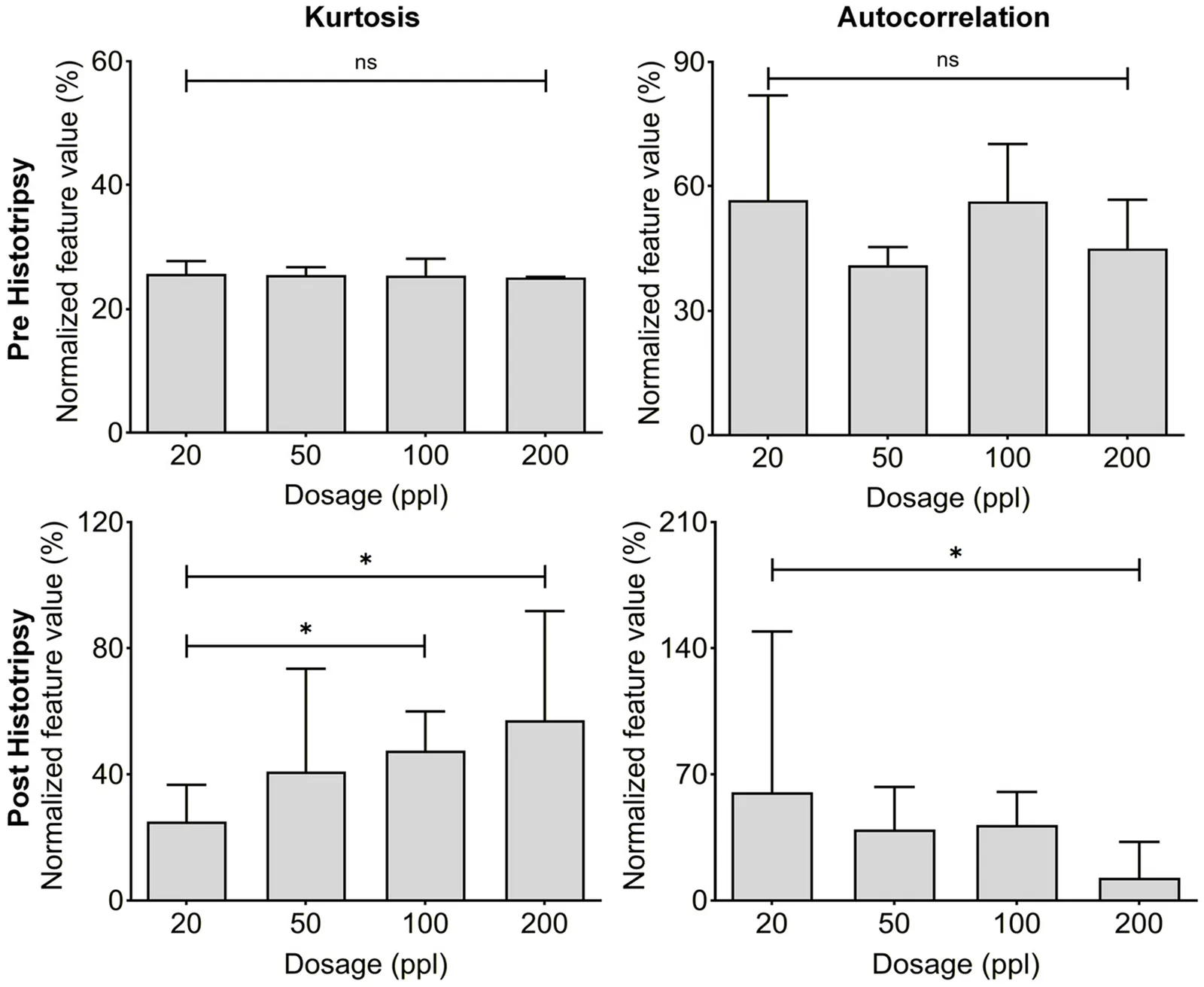
Dose dependence of the selected texture features for both pre- (top row) and post-histotripsy treatment (bottom row). Results are shown as mean ± standard deviation (SD) for Kurtosis (left column) and Autocorrelation (right column). Before treatment, neither feature showed statistically significant differences across dose groups. After treatment, both features exhibited significant dose-dependent changes at certain levels. Specifically, pairwise comparisons revealed that Kurtosis differed significantly between 20 and 100 ppl, as well as between 20 and 200 ppl (* denotes *p* < 0.05). Autocorrelation also showed a significant dose effect between 20 and 200 ppl.

Changes across all animals with increasing histotripsy dose are summarized in Figure 5 and Table 1, with values reported as pre- versus post-treatment measurements. Before treatment, neither feature showed statistically significant differences across dose groups. Kurtosis increased following histotripsy in a dose-dependent manner, with minimal change at 20 ppl and progressively larger increases at higher doses from 50 ppl to 200 ppl. Specifically, pairwise comparisons revealed that Kurtosis differed significantly between 20 and 100 ppl, as well as between 20 and 200 ppl (* denotes p < 0.05). Autocorrelation exhibited an inverse dose-dependent response pattern. Mean autocorrelation values decreased following treatment with increasing dose, from 20 ppl to 200 ppl. Pairwise comparisons indicated that statistical significance emerged only between the 20 and 200 ppl groups. A paired t-test analysis revealed no statistically significant differences between pre- and post-treatment measurements at 20 ppl for either feature (Kurtosis or Autocorrelation). Kurtosis showed statistically significant differences from baseline at 100 and 200 ppl. In contrast, Autocorrelation showed a statistically significant deviation from baseline only at the 200 ppl.

### Histological Analysis of Ablation Zone

**Figure 6.**
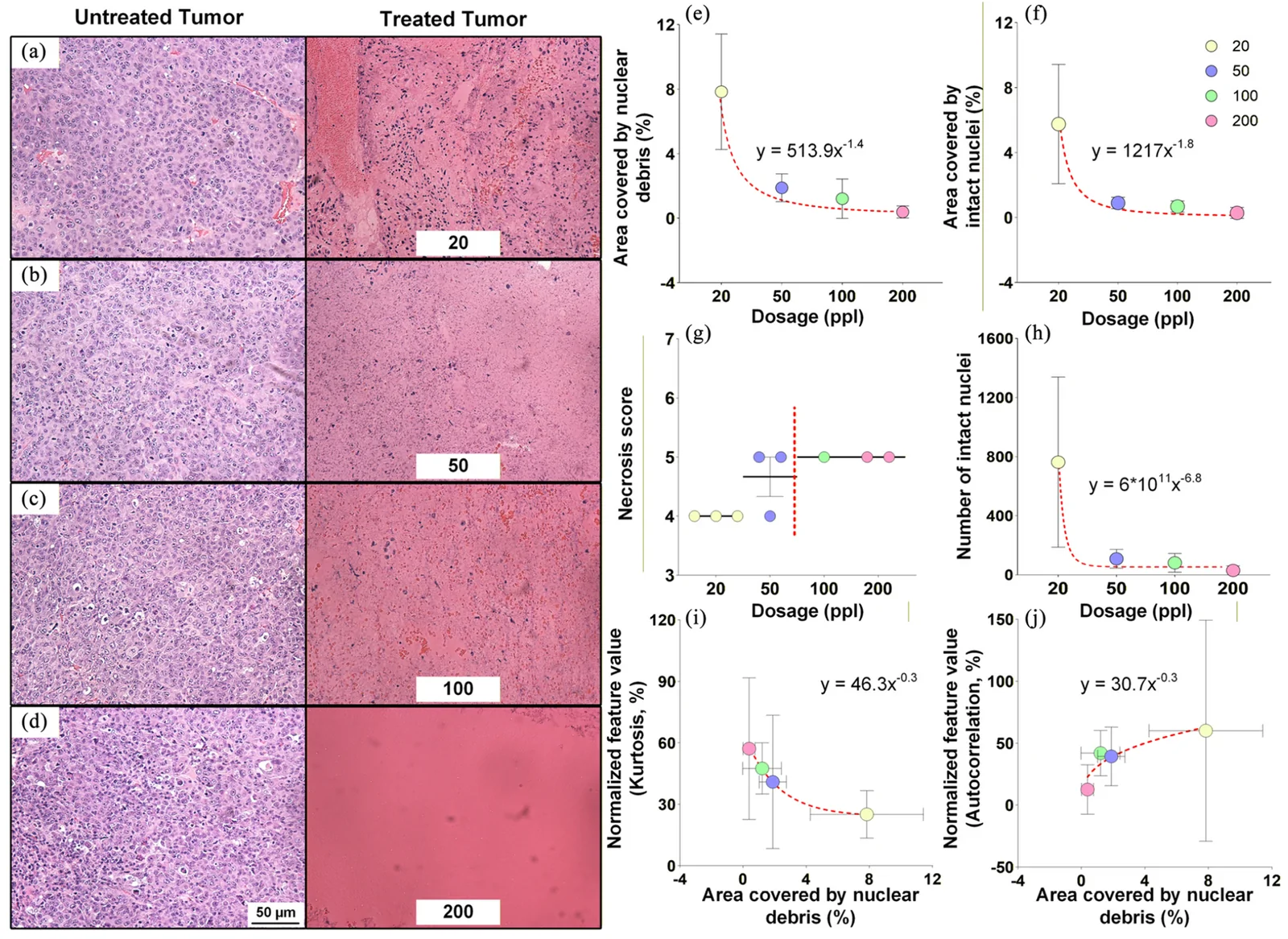
Comparison of texture analysis and histological (H&E) slides illustrating tissue structure before and after histotripsy therapy. The nuclear area fraction was calculated as the percentage of tissue occupied by hematoxylin-stained nuclei. **(a–d)** Four representative H&E sections before and after histotripsy therapy with the corresponding dosage indicated at the bottom of each image in the right column. **(e)** Shows percentage of area covered by nuclear debris as a function of histotripsy dose. **(f)** Shows the corresponding area covered by intact nuclei only. **(g)** Summary of necrosis scores for the corresponding histology images, with a red dotted line indicating the separation between partially and fully nonviable cancer cells in the treatment zone. **(h)** Plots showing the average intact nuclei counts across different dosages. **(i–j)** Correlation between imaging texture features and histology measurements, with power regression fits for the two selected features yielding R^2^ values above 0.8. Values are reported as mean ± SD.

Increased dose led to greater cellular damage within the ablation zone as confirmed by histology (Fig. 6a–d). Specifically, quantitative analysis showed that the average percentage of area covered by nuclear debris decreased as the histotripsy dose increased from 20 to 200 ppl (Fig 6e; Table 2). In addition, the percentage of area covered by intact nuclei also decreased (Fig 6f, Table 2). Necrosis scores were 4 for all rats at 20-ppl and 5 for all rats above 20-ppl, except for a single rat in the 50-ppl group that remained at 4 (Fig 6g). Consistently, the number of intact nuclei decreased with increasing dose (Fig 6h, Table 2). Subsequently, power correlation analyses revealed strong correlations between area covered by nuclear debris and both calculated kurtosis (R^2^ = 0.9) and autocorrelation (R^2^ = 0.8) (Fig 6i, j). These results suggest that histotripsy-induced cellular damage is dose-dependent and highlight the potential for these static imaging biomarkers to serve as monitors of treatment.

### Machine Learning

**Figure 7.**
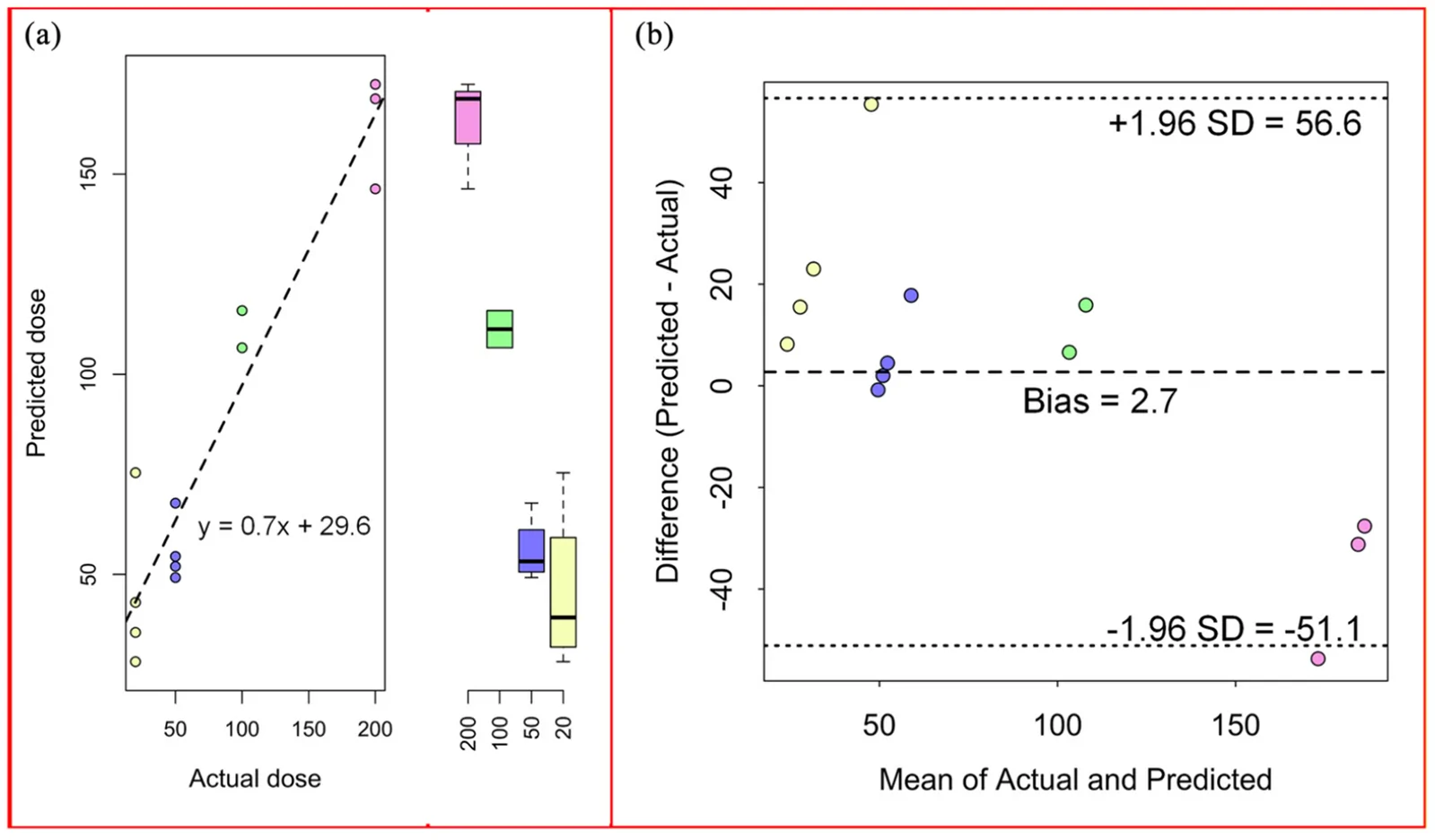
Comparison between the random forest–predicted histotripsy dose and the actual dose. **(a)** Predicted versus actual dose shows a strong linear dependence, suggesting that texture features quantitatively reflect histology-related changes in tumor microstructure across dose levels. Predictions in the 20-ppl group show greater variance than other dose groups, which may be partly due to residual tumor cell viability. Linear regression indicates a significant relationship (p < 0.05) with R^2^ = 0.9. **(b)** Agreement between predicted and actual dose is shown using a difference-versus-mean plot (predicted - actual vs mean of predicted and actual dose), which visualizes bias and the distribution of prediction errors across the dose range.

Finally, these two selected features were used to train a random forest classification model, with treatment dose as the prediction target, yielding a test R^2^ value of 0.9 with p < 0.05 (Fig 7a). In addition, agreement between the predicted and actual doses was evaluated using a Bland–Altman plot (Fig 7b). This indicates that texture features can provide quantitative insights into dose-dependent post-histotripsy changes within the tumor at a cellular level.

## DISCUSSION

Histotripsy pulses generate controlled acoustic cavitation at the focus to disrupt the targeted tumor cells noninvasively. Current clinical practice in liver tumor histotripsy ablation relies on a single, predetermined high dose of ultrasound pulses for all liver tumor types, with the aim of maximizing tumor destruction. However, tumor susceptibility to histotripsy varies based on histological and mechanical properties (15,28,29), indicating that a uniform dosing approach may not yield optimal results across different tumor types. Recent studies have demonstrated that histotripsy-induced cellular damage is dose-dependent (30–32), which can impact immune response (17) and survival outcomes (18). These findings emphasize the potential necessity for personalized dosing strategies guided by quantitative imaging feedback, rather than a one-size-fits-all approach.

In this study, we developed a quantitative imaging framework that integrated ultrasound-based texture analysis with a machine learning classification model. A range of histotripsy doses from 20 ppl to 200 ppl per location was used to cover sparse treatment to overtreatment and determine if machine learning-based texture analysis of ultrasound images could accurately reflect the degree of tissue disruption seen on histology. Our results showed clear dose-dependent changes in ultrasound texture features post- vs. pre-histotripsy, with increasing magnitude of change as treatment dose increased. These texture variations were consistent with histological evidence of progressive cellular disruption and tissue breakdown. Additionally, the nonlinear relationship between imaging features and histotripsy dose highlights the need for quantitative biomarkers for dose calibration rather than reliance on qualitative thresholds or clinical judgment. This result also aligns with previous studies that have shown changes in necrosis scores when the acoustic dose is varied (29). While higher doses demonstrated closer agreement between predicted and administered values (Fig 7), greater variability was observed at lower doses (e.g., 20 ppl). The variability observed at lower doses likely reflects early biological responses within the treated tissue, including heterogeneous patterns of cellular disruption, localized inflammation, and evolving cellular activity in the immediate post-treatment phase. The potential complexity inherent in biological tissue remodeling following histotripsy requires more in-depth study to fully understand. Future studies could investigate whether and how immune cell infiltration, inflammatory, and vascular changes within the 24-hour timeframe following histotripsy influence tissue texture results and contribute to the variability observed at lower doses to help refine dose predictions.

From a technical perspective, texture-based metrics can provide quantitative assessments of tissue microstructure that might not be possible with conventional B-mode ultrasound imaging techniques (33–35). Additionally, most current clinical ultrasound imaging assessments are reader-dependent, which can introduce variability and inconsistencies. Collectively, these issues highlight the importance of establishing objective and reproducible imaging metrics that can consistently quantify histotripsy-related tissue effects. Our study demonstrates sensitivity to dose-dependent tumor destruction and highlights the feasibility of using noninvasive imaging biomarkers for monitoring treatment effects. The approach developed in this study may allow clinicians to tailor histotripsy dosing to individual tumor characteristics, improving ablation efficiency and overall treatment outcomes. Furthermore, as histotripsy is increasingly being applied to tumors in other organs, this method may also help guide the selection of optimal treatment doses across a range of cancers. Ultimately, integration of real-time, quantitative imaging feedback into clinical practice holds promise for advancing the precision, safety, and efficacy of the noninvasive histotripsy treatments.

Our study has several limitations. First, the quantification of nuclear debris relies heavily on standardized histological staining protocols and imaging parameters, which may introduce variability. In addition, the relationship between nuclear debris and imaging features can be mathematically defined, providing a theoretical basis for prediction. Because nuclear debris is primarily used to determine treatment outcomes, predicting nuclear debris from imaging features is a promising direction for future studies. However, the limited sample size in the current study made it challenging for machine learning models to accurately estimate nuclear debris. Second, precise spatial co-registration between ultrasound images and histological sections remains challenging, complicating direct feature-to-histology comparisons. Third, smaller nuclear structures near the detection limit of histological resolution may be misclassified as complete disruption, highlighting the need for further validation to improve study reliability. Moreover, the exploratory nature of this feasibility study, with limited sample size and number of images per animal, constrains generalizability. Extending the analysis to entire tumor volumes may provide more comprehensive insight into intra- and intertumoral heterogeneity and strengthen confidence in these findings. In addition, ultrasound images were acquired using a standardized protocol, and the reliability of extracted features under different imaging settings (e.g., gain or frequency) was not evaluated. Our feature-reduction approach retains one representative feature from each highly correlated pair. While this approach may not always select the single feature that best represents tissue microstructural changes, this limitation is mitigated because the retained feature from each correlated pair contains similar information and is considered equivalent. Future work will investigate different types of liver tumors and diffuse hepatocellular disease, such as cirrhosis and fatty liver disease, along with larger datasets and immune and molecular readouts to complement histology and further evaluate the proposed approach. Further development of machine-learning models, including convolutional neural networks, is planned to enhance feature extraction and to study the relationship between histotripsy dosage, tissue damage, and ultrasound feature changes in order to improve predictive capability. Current analysis relies on manually annotated ROIs, and future studies will incorporate motion-tracking methods to account for respiratory motion in clinical data processing.

The proposed approach enables assessment of treatment efficacy beyond subjective evaluation and can detect localized changes using imaging biomarkers. These biomarkers may help inform dose adjustments across different tumor types or patient subgroups. Imaging texture features were correlated with histological findings by accounting for both intact nuclei and nuclear debris. Feature selection was derived from a subset of histology slides and applied to the full dataset for dose prediction, with repeated analyses used to reduce bias. Future work will focus on improving outcome evaluation by incorporating immune-related data and validating the approach with larger histology datasets.

## CONCLUSION

This study shows that integrating ultrasound texture analysis with machine learning offers a potential method to quantitatively evaluate histotripsy-induced tissue changes in liver tumors. By linking imaging features with histology, our approach provides a quantitative framework for non-invasively assessing imaging biomarkers associated with tissue structural changes. Importantly, the results demonstrate a clear relationship between the treatment dose and tissue response, suggesting these ultrasound metrics could help guide and fine-tune histotripsy dosing. While more research is needed to confirm these findings, this approach represents a promising step toward more personalized and effective histotripsy treatment for liver cancer patients.

## Figures and Tables

**TABLE 1.** Changes in Features Across Histotripsy Doses

Feature	Timepoint	20 ppl	50 ppl	100 ppl	200 ppl
Kurtosis	Pre-Histotripsy	25.6 ± 2.1	25.4 ± 1.3	25.3 ± 2.7	25.0 ± 0.3
	Post-Histotripsy	25.1 ± 11.6	40.9 ± 32.6	47.5 ± 12.5	57.2 ± 34.6
Autocorrelation	Pre-Histotripsy	56.6 ± 55.3	40.9 ± 9.8	56.3 ± 31.1	44.9 ± 26.4
	Post-Histotripsy	60.1 ± 89.2	39.4 ± 23.7	41.9 ± 18.3	12.6 ± 19.9

Note: Values are reported as mean ± SD.

**TABLE 2.** Changes in Cellular Damage Across Histotripsy Doses

	20 ppl	50 ppl	100 ppl	200 ppl
Percentage of area covered by nuclear debris	7.8 ± 3.6	1.8 ± 0.9	1.1 ± 1.2	0.4 ± 0.4
Percentage of area covered by intact nuclei	5.8 ± 3.7	0.9 ± 0.4	0.7 ± 0.3	0.3 ± 0.3
Intact nuclei count	763.6 ± 575.9	109.4 ± 63.9	81.9 ± 63.9	30.4 ± 31.5
Necrosis Score	4	(4, 5)*	5	5

Note: Values are reported as mean ± SD. The necrosis scores were 4 for all rats at 20-ppl, and 5 for all rats at 100 ppl and 200 ppl.

* Necrosis score was 5 for all rats at 50 ppl except for a single rat that remained at 4.

## Data Availability

Data from this study will be available upon reasonable request to the corresponding author.

## Take-home message:

- Ultrasound texture analysis combined with machine learning was used to non-invasively quantify histotripsy-induced tumor tissue damage, validated against histology.
- Imaging features derived from the post-histotripsy ablation zone demonstrated a clear histotripsy dose–response relationship, which can be used for non-invasive treatment assessment and support personalized therapy in future.

## References

[1] Marcellin P, Kutala BK. Liver diseases: a major, neglected global public health problem requiring urgent actions and large-scale screening. Liver Int 2018; 38:2–6. 29427496 10.1111/liv.13682

[2] Giorgio A, Merola MG, Montesarchio L, Percutaneous radiofrequency ablation of hepatocellular carcinoma in cirrhosis: analysis of complications in a single centre over 20 years. Br J Radiol 2017; 90:20160804. 28402124 10.1259/bjr.20160804 PMC5602175

[3] Kulik L, El-Serag HB. Epidemiology and management of hepatocellular carcinoma. Gastroenterology 2019; 156:477–491. 30367835 10.1053/j.gastro.2018.08.065 PMC6340716

[4] Rossi L, Zoratto F, Papa A, Current approach in the treatment of hepatocellular carcinoma. World J Gastrointest Oncol 2010; 2:348–359. 21160806 10.4251/wjgo.v2.i9.348 PMC2999141

[5] Ding J, Jing X, Wang Y, Wang F, Wang Y, Du Z. Thermal ablation for hepatocellular carcinoma: a large-scale analysis of long-term outcome and prognostic factors. Clin Radiol 2016; 71:1270–1276. 27510559 10.1016/j.crad.2016.07.002

[6] Maeda M, Saeki I, Sakaida I, Complications after radiofrequency ablation for hepatocellular carcinoma: a multicenter study involving 9,411 Japanese patients. Liver Cancer 2020; 9:50–62. 32071909 10.1159/000502744 PMC7024979

[7] Zavaglia C, Mancuso A, Foschi A, Rampoldi A. High-intensity focused ultrasound (HIFU) for the treatment of hepatocellular carcinoma: is it time to abandon standard ablative percutaneous treatments? Hepatobiliary Surg Nutr 2013; 2:184–187. 24570943 10.3978/j.issn.2304-3881.2013.05.02 PMC3924684

[8] Hall TL, Fowlkes JB, Cain CA. A real-time measure of cavitation induced tissue disruption by ultrasound imaging backscatter reduction. IEEE Trans Ultrason Ferroelectr Freq Control 2007; 54:569–575. 17375825 10.1109/tuffc.2007.279

[9] Khokhlova TD, Monsky WL, Haider YA, Maxwell AD, Wang Y-N, Matula TJ. Histotripsy liquefaction of large hematomas. Ultrasound Med Biol 2016; 42:1491–1498. 27126244 10.1016/j.ultrasmedbio.2016.01.020 PMC4899253

[10] Vlaisavljevich E, Kim Y, Allen S, Image-guided noninvasive ultrasound liver ablation using histotripsy: feasibility study in an In Vivo porcine model. Ultrasound Med Biol 2013; 39:1398–1409. 23683406 10.1016/j.ultrasmedbio.2013.02.005 PMC3709011

[11] Worlikar T, Vlaisavljevich E, Gerhardson T, Histotripsy for non-invasive ablation of hepatocellular carcinoma (HCC) tumor in a subcutaneous Xenograft murine model. Annu Int Conf IEEE Eng Med Biol Soc 2018:6064–6067. 30441719 10.1109/EMBC.2018.8513650

[12] Worlikar T, Mendiratta-Lala M, Vlaisavljevich E, Effects of histotripsy on local tumor progression in an in vivo orthotopic rodent liver tumor model. BME Front 2020; 2020:9830304. 34327513 10.34133/2020/9830304 PMC8318009

[13] Qu S, Worlikar T, Felsted AE, Non-thermal histotripsy tumor ablation promotes abscopal immune responses that enhance cancer immunotherapy. J Immunother Cancer 2020; 8:e000200. 31940590 10.1136/jitc-2019-000200 PMC7057529

[14] Vidal-Jové J, Serres-Créixams X, Ziemlewicz TJ, Cannata JM. Liver histotripsy mediated abscopal effect—case report. IEEE Trans Ultrason Ferroelectr Freq Control 2021; 68:3001–3005. 34310299 10.1109/TUFFC.2021.3100267

[15] Hendricks-Wenger A, Weber P, Simon A, Histotripsy for the treatment of cholangiocarcinoma liver tumors: in vivo feasibility and ex vivo dosimetry study. IEEE Trans Ultrason Ferroelectr Freq Control 2021; 68:2953–2964. 33856990 10.1109/TUFFC.2021.3073563 PMC9297335

[16] Zhong X, Long H, Chen L, Stiffness on shear wave elastography as a potential microenvironment biomarker for predicting tumor recurrence in HBV-related hepatocellular carcinoma. Insights Imaging 2023; 14:147. 37697029 10.1186/s13244-023-01505-7 PMC10495298

[17] Tang S, McGinnis R, Cao Z, Baker JR, Xu Z, Wang S. Ultrasound-guided histotripsy triggers the release of tumor-associated antigens from breast cancers. Cancers 2025; 17:183. 39857965 10.3390/cancers17020183 PMC11764245

[18] McGinnis R, Song B, Kim H, Histotripsy dose impacts treated tumor immune infiltration and survival outcomes in a murine B16F10 melanoma model. Cancers 2025; 17:3773. 41374975 10.3390/cancers17233773 PMC12691148

[19] Worlikar T, Zhang M, Ganguly A, Impact of histotripsy on development of intrahepatic metastases in a rodent liver tumor model. Cancers 2022; 14:1612. 35406383 10.3390/cancers14071612 PMC8996987

[20] Rueden CT, Schindelin J, Hiner MC, ImageJ2: ImageJ for the next generation of scientific image data. BMC Bioinform 2017; 18:529. 10.1186/s12859-017-1934-z PMC5708080 29187165

[21] Weigert M, Schmidt U. Nuclei instance segmentation and classification in histopathology images with stardist. 2022 IEEE Int Symp Biomed Imaging Chall 2022:1–4.

[22] Tai H, Kalayeh K, Ashton-Miller JA, DeLancey JO, Brian Fowlkes J. Urethral tissue characterization using multiparametric ultrasound imaging. Ultrasonics 2025; 145:107481. 39348748 10.1016/j.ultras.2024.107481 PMC12789978

[23] Tai H, Pipitone F, Kalayeh K, Assessing postpartum levator ani muscle recovery: a feasibility study on automated texture analysis in transvaginal ultrasound. Ultrasound Med Biol 2025; 51:2217–2225. 40976767 10.1016/j.ultrasmedbio.2025.08.024 PMC12805553

[24] van Griethuysen JJM, Fedorov A, Parmar C, Computational radiomics system to decode the radiographic phenotype. Cancer Res 2017; 77:e104–e107. 29092951 10.1158/0008-5472.CAN-17-0339 PMC5672828

[25] Grömping U. Variable importance assessment in regression: linear regression versus random forest. The American Statistician 2009; 63:308–319.

[26] Wang T-Y, Xu Z, Winterroth F, Quantitative ultrasound backscatter for pulsed cavitational ultrasound therapy-histotripsy. IEEE Trans Ultrason Ferroelectr Freq Control 2009; 56:995–1005. 19750596 10.1109/tuffc.2009.1131 PMC3130252

[27] Jin W, Tian Y, Xuzhang W, Non-linear modifications enhance prediction of pathological response to pre-operative PD-1 blockade in lung cancer: a longitudinal hybrid radiological model. Pharmacol Res 2023; 198:106992. 37977237 10.1016/j.phrs.2023.106992

[28] Vlaisavljevich E, Kim Y, Owens G, Roberts W, Cain C, Xu Z. Effects of tissue mechanical properties on susceptibility to histotripsy-induced tissue damage. Phys Med Biol 2014; 59:253–270. 24351722 10.1088/0031-9155/59/2/253 PMC4888779

[29] Vlaisavljevich E, Maxwell A, Warnez M, Johnsen E, Cain CA, Xu Z. Histotripsy-induced cavitation cloud initiation thresholds in tissues of different mechanical properties. IEEE Trans Ultrason Ferroelectr Freq Control 2014; 61:341–352. 24474139 10.1109/TUFFC.2014.6722618 PMC4158820

[30] Wang T, Xu Z, Winterroth F, Quantitative ultrasound backscatter for pulsed cavitational ultrasound therapy- histotripsy. IEEE Trans Ultrason Ferroelectr Freq Control 2009; 56:995–1005. 19750596 10.1109/tuffc.2009.1131 PMC3130252

[31] Haskell SC, Yeats E, Shi J, Acoustic cavitation emissions predict near-complete/complete histotripsy treatment in soft tissues. Ultrasound Med Biol 2025; 51:909–920. 40015999 10.1016/j.ultrasmedbio.2025.02.005 PMC11925334

[32] Macoskey JJ, Zhang X, Hall TL, Bubble-induced color doppler feedback correlates with histotripsy-induced destruction of structural components in liver tissue. Ultrasound Med Biol 2018; 44:602–612. 29329687 10.1016/j.ultrasmedbio.2017.11.012 PMC5801099

[33] Rathore S, Iftikhar MA, Hussain M, Jalil A. Texture analysis for liver segmentation and classification: a survey. 2011 Front Inform Technol 2011; 121:126.

[34] Das A, Connell M, Khetarpal S. Digital image analysis of ultrasound images using machine learning to diagnose pediatric nonalcoholic fatty liver disease. Clin Imaging 2021; 77:62–68. 33647632 10.1016/j.clinimag.2021.02.038

[35] Tai H, Song J, Li J, Reddy S, Khairalseed M, Hoyt K. Three-dimensional H-scan ultrasound imaging of early breast cancer response to neoadjuvant therapy in a murine model. Investig Radiol 2022; 57:222–232. 34652291 10.1097/RLI.0000000000000831 PMC8916970

